# Differential Serotonergic Modulation of Synaptic Inputs to the Olfactory Cortex

**DOI:** 10.3390/ijms24031950

**Published:** 2023-01-19

**Authors:** Ildikó Piszár, Magor L. Lőrincz

**Affiliations:** 1 Department of Physiology, Anatomy and Neuroscience, University of Szeged, 6726 Szeged, Hungary; 2 Department of Physiology, University of Szeged, 6720 Szeged, Hungary; 3 Neuroscience Division, Cardiff University, Cardiff CF10 3AX, UK

**Keywords:** neuromodulation, synaptic transmission, interneuron, olfactory cortex

## Abstract

Serotonin (5-hydroxytriptamine, 5-HT) is an important monoaminergic neuromodulator involved in a variety of physiological and pathological functions. It has been implicated in the regulation of sensory functions at various stages of multiple modalities, but its mechanisms and functions in the olfactory system have remained elusive. Combining electrophysiology, optogenetics and pharmacology, here we show that afferent (feed-forward) pathway-evoked synaptic responses are boosted, whereas feedback responses are suppressed by presynaptic 5-HT_1B_ receptors in the anterior piriform cortex (aPC) in vitro. Blocking 5-HT_1B_ receptors also reduces the suppressive effects of serotonergic photostimulation of baseline firing in vivo. We suggest that by regulating the relative weights of synaptic inputs to aPC, 5-HT finely tunes sensory inputs in the olfactory cortex.

## 1. Introduction

The ability to adapt to an ever-changing environment is key to survival. Sensory systems constantly extract and process signals from both the external world and internal sources to form representations of the environment. This is ultimately used to fine-tune a dynamic behavioral repertoire according to current or anticipated ecological contexts, which is a process that occurs on various timescales and is influenced by previous experience. Various sensory functions are affected by neuromodulators, such as the monoamines. These crucial molecules are present at the peripheral, intermediate, and central stages of every sensory modality. In addition to the widespread monoaminergic innervation of sensory pathways, monoaminergic neurons receive sensory inputs from various modalities [1], resulting in reciprocal interaction between sensory and neuromodulatory systems.

Located in the brainstem raphé nuclei (RN), serotonergic neurons project to various forebrain areas and release serotonin (5-hydroxytryptamine, 5-HT) throughout the entire neuroaxis. 5-HT is implicated in a variety of physiological functions, including the regulation of sensory and motor responses [2,3,4,5], brain states [6,7,8], learning and reward processing [9,10,11], fear responses [12] and social interactions [13,14]. Dysfunctions of the serotonergic system are implicated in several neurological and psychiatric disorders, including depression [15] and epilepsy [16,17]. RN 5-HT fibers densely innervate the primary olfactory cortex (aPC, anterior piriform cortex) with both the dorsal raphe nucleus (DRN) and median raphe nucleus (MRN) neurons contributing to this projection [18]. The most prominent effect of exogenously applied 5-HT in the aPC is a 5HT_1A_ receptor-mediated hyperpolarization of principal neurons [19] and a 5HT_2_ and 5HT_3_ receptor-mediated depolarization of local interneurons [20,21,22,23], which coincides with an increase in inhibitory postsynaptic potentials (IPSPs) in principal neurons [24]. Interestingly, the local photostimulation of 5-HT axons has little effect on their membrane potential [25], but can reduce the excitability of principal neurons in the aPC [25,26] and lead to a 5HT_2A_ receptor-mediated membrane potential depolarization and a subsequent increase in action potential output in interneurons, including perisomatic inhibitory fast-spiking GABAergic neurons [25]. Importantly, the specific stimulation of DRN 5-HT neurons results in a prominent suppression of spontaneous but not odor-evoked activity of most aPC neurons in vivo [2], but the mechanisms involved in this differential action have remained elusive. 

Here, we address this question by combining electrophysiology, optogenetics and pharmacology and show that 5-HT can increase responses evoked by feed-forward inputs originating from the OB but suppress feedback inputs originating from various cortical sources. The suppression of feedback synaptic inputs was due to the modulation of glutamate release via presynaptic 5-HT_1B_ receptors. By this input-specific effect, 5-HT contributes to the fine tuning of sensory computations in general and to olfactory ones in particular.

## 2. Results

**Figure 1:**
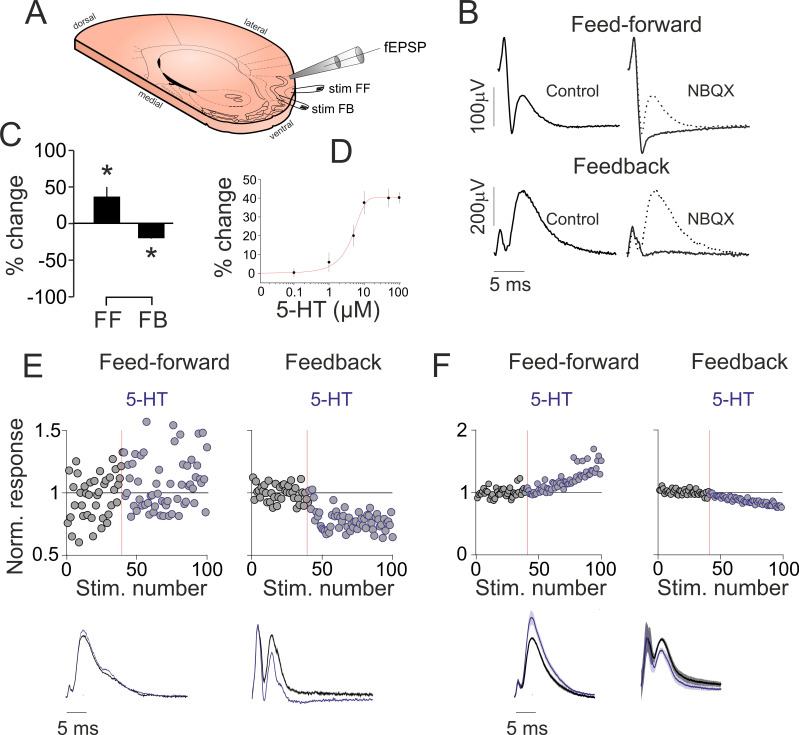
**5-HT suppresses intracortical but increases afferent synaptic activity in vitro.** (**A**) Schematics of the experimental design: tilted, angled coronal section of the brain showing the placement of the stimulating and recording electrodes. (**B**) Stimulation of the LOT (top, left) and aPC layer 2 (bottom, left) results in both volley spikes and field excitatory postsynaptic potentials (fEPSPs). The fEPSPs are blocked by the AMPA/KA glutamate receptor blocker NBQX (right). (**C**) Bar graph illustrating the 5-HT-induced changes in LOT and layer 2 stimulation-evoked fEPSPs in 8 recordings (values correspond to average peak responses during the last 20 trials following 5-HT application). (**D**) Dose–response curve of the layer 2 evoked fEPSPs. (**E**) (Top) Single experiment time course of the fEPSP slope changes by 5-HT for feed-forward and feedback pathway stimulation. Application of 5-HT (blue traces, 10 µM in the perfusing solution) increasesed LOT stimulation-evoked (left, feed-forward), but suppressed layer 2 evoked fEPSPs (right, feedback). Average fEPSPs recorded from aPC layer 2b following either LOT (left, feed-forward) or layer 2 stimulation (right, feedback). (**F**) (Top) Grand-average (n = 8 recordings) time course of the fEPSP slope changes by 5-HT for feed-forward and feedback pathway stimulation. The application of 5-HT resulted in the increase of LOT stimulation-evoked, but suppressed layer 2 evoked fEPSPs. (Bottom) Grand-average fEPSPs recorded from aPC layer 2b following either LOT (left, feed-forward) or layer 2 stimulation (right, feedback) from n = 8 experiments. * indicates *p* < 0.05.

**Figure 2:**
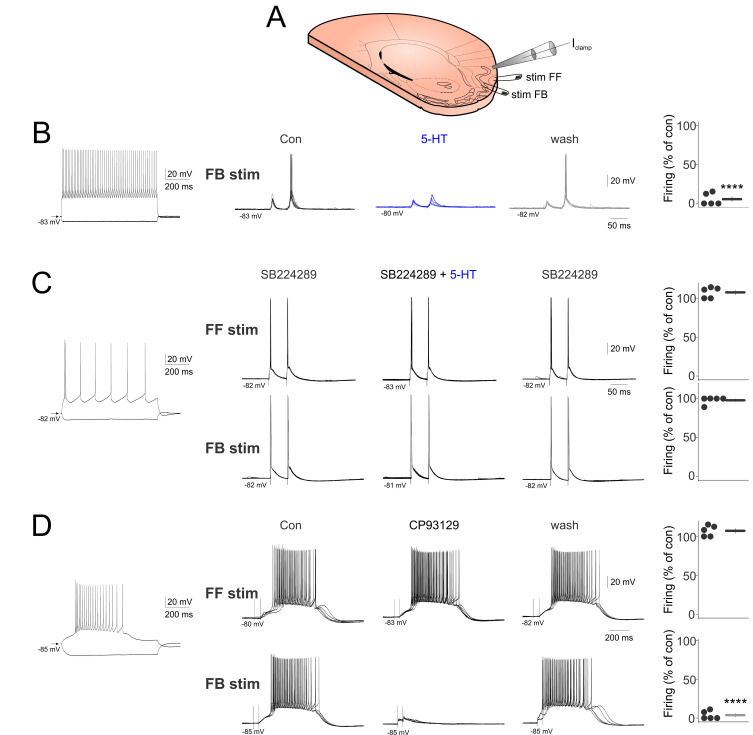
**5-HT suppresses FB stimulation-induced firing via 5-HT_1B_ receptors.** (**A**) Schematics of the experimental design. (**B**) (Left) Membrane responses to hyperpolarizing and depolarizing current pulses of a layer 3 fast spiking neuron. (Middle) Bath applied 5-HT suppresses the FB stimulation-evoked firing. (Right) Quantified effects of 5-HT on FB stimulation-induced firing for all neurons recorded (n = 5). (**C**) (Left) Membrane responses to hyperpolarizing and depolarizing current pulses of a layer 2 regular spiking neuron. (Middle) Bath applied 5-HT fails to suppress the FF and FB stimulation evoked firing in the presence of the 5-HT_1B_ receptor blocker SB224289 (10 µM). (Right) Quantified effects of 5-HT on FF and FB stimulation-induced firing in the presence of the 5-HT_1B_ receptor blocker SB224289 for all neurons recorded (n = 5). (**D**) (Left) Membrane responses to hyperpolarizing and depolarizing current pulses of an L1 interneuron. (Middle) Bath applied CP93129 (10 µM) suppresses the FB but not FF stimulation-evoked firing. (Right) Quantified effects of CP93129 on FF and FB stimulation-induced firing for all neurons recorded (n = 5). **** indicates *p* < 0.0001.

We first scrutinized the effects of 5-HT on various synaptic inputs to the aPC in vitro (Figure 1A). The brief (0.1 ms) electrical stimulation of LOT resulted in feed-forward (FF) field excitatory postsynaptic potentials (fEPSPs), while stimulating aPC layer 2 resulted in feedback (FB) fEPSPs (Figure 1B left), as earlier shown [27,28,29]. Both fEPSPs could be blocked by the AMPA/KA glutamate receptor blocker NBQX (10 µM), while the axonal volley persisted (Figure 1B right; n = 3 slices). We normalized individual FF and FB fEPSPs to the magnitude of the fiber volley and quantified the effect of bath applied 5-HT (10 µM) on the fEPSPs. The peak of the FF fEPSPs was increased by 5-HT (5-HT: +37 ± 0.13%; n = 8 slices; *p* < 0.05, Figure 1C), while FB fEPSPs were suppressed (5-HT: −19 ± 0.04% of control; n = 8 slices; *p* < 0.01, Figure 2C), suggesting 5-HT has a pathway specific effect on aPC synaptic responses. As 10 µM 5-HT yielded near maximal effects (Figure 1D), we used this concentration throughout this study. Figure 1E illustrates a single experiment time course of the 5-HT effects on FF and FB inputs. Figure 1F shows that the grand average time course of the fEPSP slope changes by 5-HT for FF and FB pathway stimulation. The 5-HT_1_ receptor agonist 5-carboxamidotryptamine maleate (5-CT, 50 nM) could also suppress FB fEPSPs (−29.71 ± 2.37%, *p* = 0.006, data not illustrated) with a simultaneous increase in paired pulse ratio (con: 1.07 ± 0.06%, CT: 1.25 ± 0.09%, *p* < 0.05), suggesting presynaptic 5-HT_1A_ or 5-HT_1B_ receptors could be mediating the observed effects.

We next studied the effect of 5-HT on the synaptic stimulation effects of individual aPC neurons. To this end, we recorded various aPC neurons in whole-cell current clamp mode and set the FB and/or FF stimulation intensity to evoke moderate action potential firing. Figure 2B shows an example layer 3 fast spiking neuron that ceased FB pathway-evoked firing following 5-HT application. The suppression of FB stimulation-induced firing by 5-HT was significant for the population of neurons recorded (number of spikes evoked in control: 0.94 ± 0.10, number of spikes evoked in 5-HT: 0.06 ± 0.04, *p* < 0.001). This corresponded to a reduction in firing to 5.57 ± 3.44% of control (n = 5, *p* < 0.0001, Figure 2B right). The effect was reversed by washing out 5-HT from the perfusion chamber. To reveal the receptor type involved in mediating the suppression of firing, we blocked 5-HT_1B_ receptors by bath application of 10 µM SB224289 and scrutinized the effect of 5-HT on aPC neuronal firing evoked by FF and FB synaptic stimulation, respectively. In the presence of 5-HT_1B_ receptor blockers, 5-HT failed to suppress synaptic FF (spikes evoked by FF stimulation in SB: 0.84 ± 0.05, spikes evoked FF stimulation in SB + 5-HT: 0.9 ± 0.044, n = 5, *p* > 0.05), corresponding to a reduction in firing of 7.57 ± 3.134%, n = 5, *p* > 0.05, Figure 2C) or FB stimulation-induced firing (spikes evoked by FB stimulation in SB: 0.90 ± 0.07, spikes evoked FB stimulation in SB + 5-HT: 0.88 ± 0.073, n = 5, *p* > 0.05), corresponding to a change in firing of 2.33 ± 2.22% (n = 5, *p* > 0.05, Figure 2C bottom).

The 5-HT_1B_ receptor agonist, CP93129 (10 µM), replicated the suppressive effects of 5-HT for FB (spikes evoked by FB stimulation in control: 4.3 ± 3.22, spikes evoked by FB stimulation in CP93129: 0.04 ± 0.02, n = 5, *p* < 0.00001), leading to a reduction of 97.24% of control (n = 5, *p* < 0.00001, Figure 2D bottom) but not FF stimulation (spikes evoked by FF stimulation in control: 3.68 ± 2.68, spikes evoked by FF stimulation in CP93129: 4.16 ± 3.11, n = 5, *p* > 0.05), leading to an increase of 7.22 ± 3.14% of control (n = 5, *p* > 0.05); see Figure 2D top. These results suggest 5-HT can suppress FB responses by acting on 5-HT_1B_ receptors.

**Figure 3:**
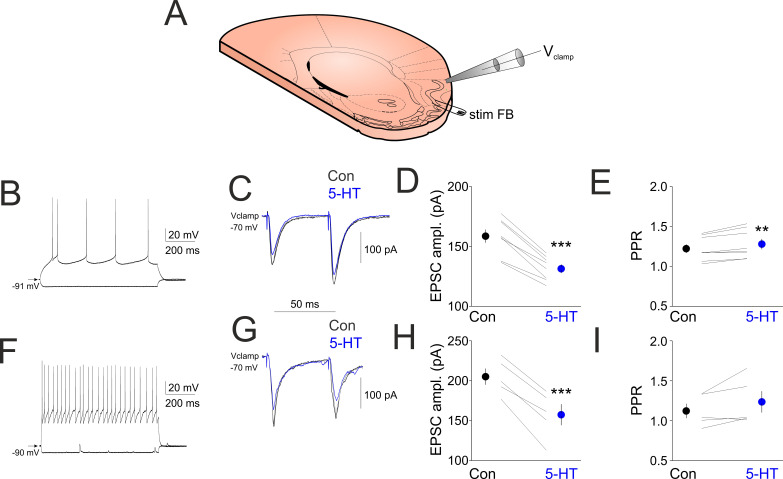
**5-HT suppresses FB stimulation induced synaptic currents.** (**A**) Schematics of the experimental design. (**B**) Membrane responses of a layer 2 pyramidal neuron. (**C**) Synaptic currents evoked by electrical stimulation of layer 2 in control conditions (black line) in the presence of 10 µM 5-HT (blue line) and following washout of 5-HT (gray line). (**D**) Quantification of EPSC_1_ amplitude changes following bath application of 5-HT in all principal neurons recorded (n = 8). (**E**) Changes in paired pulse ratios following 5-HT application in all principal neurons recorded (n = 8). (**F**) Membrane responses of a layer 3 interneuron. (**G**) Synaptic currents evoked by electrical stimulation of layer 2 in control conditions (black line) in the presence of 10 µM 5-HT (blue line) and following washout of 5-HT (gray line). (**H**) Quantification of EPSC_1_ amplitude changes following bath application of 5-HT in all interneurons recorded (n = 5). (**I**) Changes in paired pulse ratios following 5-HT application in all interneurons recorded (n = 5). ** indicates *p* < 0.01, *** indicates *p* < 0.001.

We next revealed the effects of 5-HT on excitatory postsynaptic currents (EPSCs) evoked by layer 2 electrical stimulation in various aPC neurons in whole-cell voltage clamp mode. In aPC principal neurons (Figure 3B), two brief electrical stimuli (0.1 ms, 20–100 µA) delivered to layer 2 evoked clear EPSCs that were reduced in amplitude following the bath application of 10 µM 5-HT (EPSC_1_ control: 158.75 ± 5.35, EPSC_1_ 5-HT 131.5 ± 3.48, *p* < 0.001, EPSC_2_ control: 192.87 ± 7.09, EPSC_2_ 5HT: 167.0 ± 5.15, n = 5, *p* < 0.001, Figure 3C,D). The paired pulse ratio (PPR, EPSC_2_/EPSC_1_) was significantly increased following 5-HT application (PPR control: 1.22 ± 0.05, PPR 5HT: 1.27 ± 0.06, n = 5, *p* < 0.01, Figure 3E). In aPC interneurons (Figure 3F), EPSCs were also reduced in amplitude following bath application of 5-HT (EPSC_1_ control: 205.2 ± 9.92, EPSC_1_ 5-HT 157.4 ± 12.98 (*p* < 0.001), EPSC_2_ control: 230.2 ± 22.18, EPSC_2_ 5HT: 191 ± 19.70, n = 5, *p* < 0.001, Figure 3G,H). The paired pulse ratio showed a tendency of increase following 5-HT application, but this was below significance level (PPR control: 11.12 ± 0.08, PPR 5HT: 1.23 ± 0.13, n = 5, *p* > 0.05, Figure 3I).

**Figure 4:**
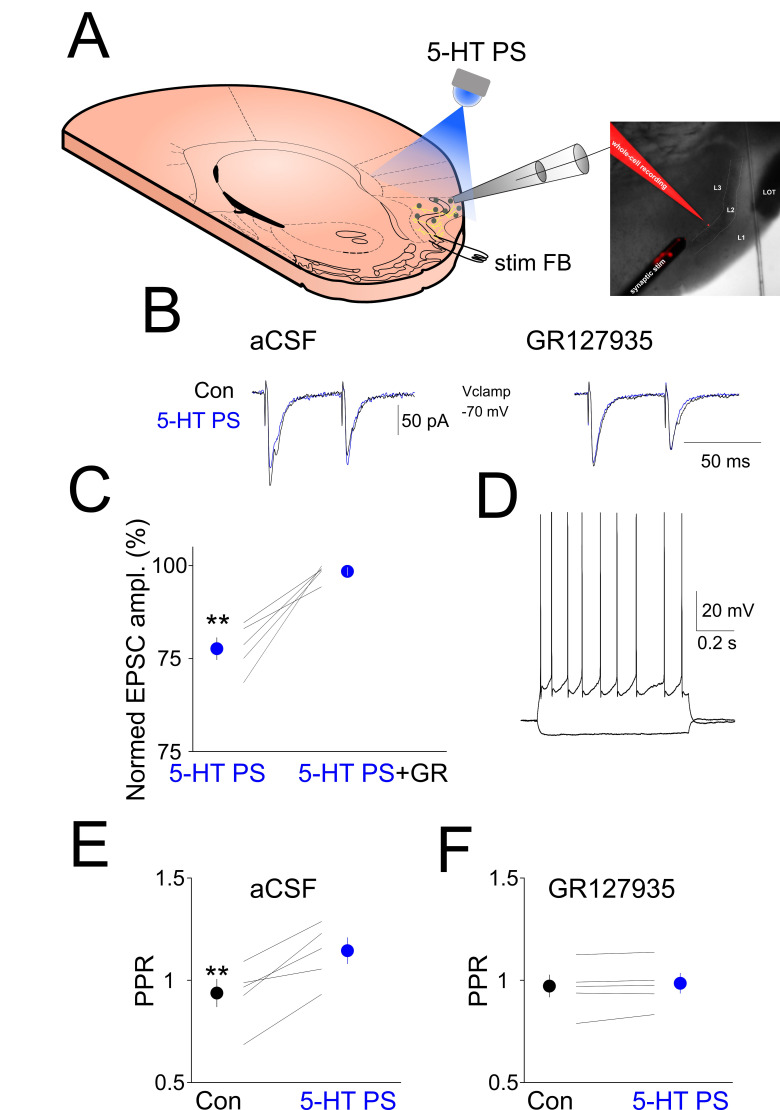
Local photostimulation of ChR2-expressing DRN axons in the aPC suppresses FB stimulation-induced synaptic currents. (**A**) Schematics of the experimental design. (**B**) (Left) Synaptic currents evoked by electrical stimulation of layer 2 are suppressed by the local photostimulation of ChR2 expressing 5-HT axons (5-HT PS, blue line) compared to control (Con, black line). (Right) The 5-HT_1B_ receptor antagonist GR127935 (10 µM) blocks the effect of 5-HT fibers photostimulation. (**C**) Quantification of the EPSC suppression by 5-HT PS in the absence and presence of 5-HT_1B_ receptor antagonist GR127935. (**D**) Membrane responses of the layer 3 pyramidal neuron shown in (**A**,**B**) to hyperpolarizing and depolarizing current pulses. (**E**) Changes in paired pulse ratio (PPR, EPSC1/EPSC2) following 5-HT PS in all the neurons recorded (n = 5). (**F**) Changes in paired pulse ratio (PPR, EPSC1/EPSC2) following 5-HT PS in the presence of 5-HT_1B_ receptor antagonist GR127935 in all the neurons recorded (n = 5). ** indicates *p* < 0.01.

To reveal the effect of endogenously released 5-HT on EPSCs evoked by local synaptic stimulation of the FB pathway, we electrically stimulated layer 2 in the absence and presence of local photostimulation of ChR2 expressing 5-HT axons (5-HT PS) while recording aPC neurons in whole-cell voltage clamp mode (Figure 4A). This evoked clear EPSCs that were reduced in amplitude following 5-HT PS (EPSC_1_ control: 234.2 ± 21.99 pA, EPSC_1_ 5-HT PS 180.4 ± 14.54, *p* < 0.01; EPSC_2_ control: 222.6 ± 34.35, EPSC_2_ 5HT PS: 207.4 ± 25.77, n = 5, *p* > 0.05, Figure 4B,C). In the presence of the selective 5-HT_1B_ receptor blocker GR127935, 5-HT PS failed to decrease the amplitude of the FB stimulation-evoked EPSCs (EPSC_1_ ampl GR127935: 213.6 ± 25.38, EPSC_1_ ampl 5-HT PS + GR127935: 211.2 ± 26.25, n = 5, *p* > 0.05, EPSC_2_ ampl GR127935: 210.6 ± 34.45, EPSC_2_ ampl 5-HT PS + GR127935: 208.4 ± 33.98, n = 5, *p* > 0.05, Figure 4B,C). The PPR was significantly increased following 5-HT PS (PPR control: 0.92 ± 0.07, PPR 5HT PS: 1.13 ± 0.06, n = 5, *p* < 0.01, Figure 4E). In the presence of GR127935, 5-HT PS failed to increase the PPR (PPR GR127935: 0.96 ± 0.05, PPR GR127935 + 5-HT PS: 0.97 ± 0.05, n = 5, *p* > 0.05, Figure 4F).

**Figure 5:**
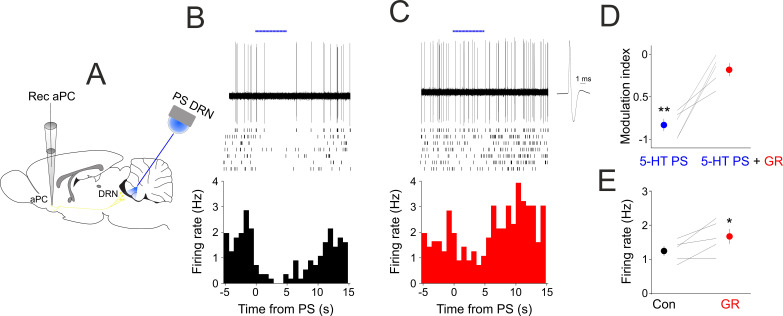
**Blocking 5-HT_1B_ receptors reduces the suppressive effect of DRN photostimulation in vivo.** (**A**) Schematics of the experimental design. (**B**) DRN photostimulation suppresses aPC baseline activity. Raw single trial example recording of an aPC neuron shows prominent suppression of its baseline action potential firing following DRN photostimulation (blue bars, 10 ms pulses at 30 Hz, 5 mW). Rasters of 7 consecutive trials are shown below. (Bottom) Peri/event time histogram of the photostimulated aPC neuron. Time zero marks the start of the photostimulation. (**C**) Same neuron as in (**B**), but following systemic administration of the 5-HT_1B_ receptor antagonist GR127935 (3 mg/kg). The action potential average is shown on a faster time-base on the right. (**D**) Modulation index in control conditions (Con) and following GR127935 administration of all aPC neurons recorded (n = 5). ** *p* < 0.01. (**E**) Baseline firing in control conditions (Con) and following GR127935 administration of all aPC neurons recorded (n = 5). * indicates *p* < 0.05.

To reveal the role of 5-HT_1B_ receptors in mediating the effects of 5-HT in vivo, we recorded the spontaneous firing of aPC neurons in anesthetized SERT-cre mice expressing ChR2 in their DRN neurons (Figure 5A). As previously shown [2], 5-HT PS lead to a significant suppression of baseline firing in all aPC neurons recorded (modulation index control: −0.83 ± 0.07, n = 5, *p* < 0.01, Figure 5B,D), but this suppressive effect of 5-HT PS was absent following the systemic administration of the 5-HT_1B_ receptor antagonistGR127935 (modulation index GR127935: −0.18 ± 0.01, n = 5, *p* > 0.05, Figure 5C,D). Blocking 5-HT_1B_ receptors also led to an increase in the baseline firing rate in the absence of 5-HT PS (FR con: 1.24 ± 0.14, FR GR127935: 1.67 ± 0.21, n = 5 neurons, *p* < 0.05, Figure 5E).

## 3. Discussion

By performing a combination of in vivo and in vitro electrophysiology, optogenetics and pharmacology, we show that (i) 5-HT can differentially affect synaptic inputs to the aPC by suppressing intracortical and increasing afferent inputs, (ii) the suppression of feedback inputs is most likely due to a 5-HT_1B_-dependent decrease in glutamate release, and (iii) the suppression of baseline aPC neuronal activity by the specific stimulation of DRN 5-HT neurons can be blocked by the systemic application of 5-HT_1B_ receptorantagonists.

These results provide a synaptic mechanism for our previous observations that 5-HT can suppress spontaneous but not odor-evoked activity [2] and complement our observations concerning the direct effect of 5-HT on single aPC principal neurons and interneurons [25]. Thus, 5-HT can directly suppress the activity of aPC principal neurons, increase the activity of aPC GABAergic interneurons and increase feed-forward synaptic inputs originating from the olfactory bulb while suppressing feedback inputs originating from various cortical sources including the aPC.

Similar to acetylcholine and noradrenaline [30,31], 5-HT can suppress feedback synaptic responses originating from cortical sources but not afferent (feed-forward) inputs originating from the OB. This differential effect on various inputs is in line with a synapse-specific effect of 5-HT, which is a general feature of this neuromodulator in various brain regions such as the olfactory tubercle [32], the hippocampus [33], and the nucleus accumbens [34].

Testing the effect of 5-HT on the spontaneous and odor-evoked activity of the aPC has led to contrasting results. The selective stimulation of DRN 5-HT neurons resulted in the divisive suppression of spontaneous, but not odor-evoked, spiking activity of anesthetized mice [2]. However, odor-evoked activity was decreased, and spontaneous activity remained unaltered during similar manipulation of 5-HT neurons when monitoring the population Ca^2+^ dynamics of aPC principal neurons using fiber photometry in awake mice [26]. Thus, the inhibition of sensory responses seems to be an important general feature of 5-HT across multiple sensory modalities, whereas its effects on spontaneous activity may vary as a function of brain region and state of vigilance.

This study has shed light on the identity of 5-HT receptors involved in the modulation of feedback inputs to the aPC. Both endogenous and exogenous application of 5-HT decreased FB stimulation induced fEPSPs and EPSCs in single neurons. In addition to the reduction in EPSCs in single aPC neurons, bath-applied 5-HT or the local photostimulation of ChR2-expressing DRN axons in the aPC led to an increase in the paired pulse ratio, suggesting a presynaptic site of action. Presynaptic 5-HT_1B_ receptors are key players in decreasing the release of glutamate in various brain areas [35,36,37,38,39,40,41]. The mechanisms of 5-HT mediated modulation of feed-forward inputs will need to be revealed by future studies.

The differential effect of 5-HT on feed-forward and feedback inputs can have important functional implications. As the afferent inputs to the aPC originate in the OB, these results argue for a locus-specific effect, where 5-HT can differentially modulate neurons and/or synapses located at various levels of the sensory pathway. 5-HT can thus regulate the relative weights of synaptic inputs to aPC influenced by the multiple synaptic input sources of the DRN [1]. One of the major sources of inhibitory and excitatory inputs to DRN neurons is the lateral hypothalamus [8,42] that can broadcast information related to energy balance and arousal to the olfactory system.

In conclusion, two important dichotomy-based motifs seem to empower the neuromodulatory function of 5-HT, its antagonistic effects on excitatory and inhibitory neurons [25], and its pathway-specific synaptic effects: generally suppressing intracortical synapses and sparing feed-forward inputs from the sensory periphery.

## 4. Materials and Methods

All experimental procedures were performed in accordance with the European Union Directive (86/609/EEC) and approved by the local ethical committees. Data are presented as mean ± SEM, unless stated otherwise.

For the selective stimulation of DRN serotonergic neurons, adult male heterozygous SERT–Cre mice [43] were injected with 0.5–1 μL of AAV2/1–Flex–ChR2–YFP (AV-1-20298P, University of Pennsylvania, 10^13^ GC/mL) in the DRN (coordinates: anteroposterior (AP), −4.7 mm; dorsoventral (DV), 3.1–3.6 mm; mediolateral (ML), 0.0 mm), leading to prominent and specific channelrhodopsin (ChR_2_) expression in DRN 5-HT neurons [2,3] and axons in the aPC [25]. Then, 8–16 weeks following the viral infections, mice were used for in vitro experiments. Mice were deeply anesthetized with ketamine and xylazine (80 and 10 mg/kg, respectively) and perfused through the heart with a solution containing the following (in mM): 93 NMDG, 2.5 KCl, 1.2 NaH_2_PO_4_, 30 NaHCO_3_, 20 HEPES, 25 glucose, 5 N-acetyl-L-cysteine, 5 Na-ascorbate, 3 Na-pyruvate, 10 MgSO_4_, and 0.5 CaCl_2_. The same solution was used to cut 320 µm coronal slices containing the aPC at 4 °C and for the initial storage of slices (32–34 °C for 12 min), following which the slices were stored in a solution containing the following (in mM): 30 NaCl, 2.5 KCl, 1.2 NaH_2_PO_4_, 1.3 NaHCO_3_, 20 HEPES, 25 glucose, 5 N-acetyl-L-cysteine, 5 Na-ascorbate, 3 Na-pyruvate, 3 CaCl_2_, and 1.5 MgSO_4_. For recording, slices were submerged in a chamber perfused with a warmed (34 °C) continuously oxygenated (95% O2, 5%CO_2_) artificial cerebrospinal fluid (ACSF) containing the following (in mM): 130 NaCl, 3.5 KCl, 1 KH_2_PO_4_, 24 NaHCO_3_, 1.5 MgSO_4_, 3 CaCl_2_, and 10 glucose. Whole-cell patch clamp recordings were performed in either current clamp or voltage clamp mode using 4–6 MOhm pipettes containing (in mM): 126 Kgluconate, 4 KCl, 4 ATP-Mg, 0.3 GTP-Na2, 10HEPES, 10 creatine-phosphate, and 8 Biocytin, pH 7.25; osmolarity, 275 mOsm. Neurons were visualized using DIC imaging on an Olympus BX51WI microscope (Tokyo, Japan). Membrane potentials and currents were recorded using a Multiclamp 700B amplifier (Molecular Devices, San Jose, CA, USA). The liquid junction potential (−13 mV) was compensated for. Series resistance was continuously monitored and compensated during the course of the experiments; recordings were discarded if the series resistance changed more than 25%. Neurons were classified as principal neurons or interneurons based on somatic morphology under DIC, membrane responses to hyperpolarizing and depolarizing current steps.

For synaptic stimulation, two concentric bipolar stimulating electrodes (FHC, Germany) were positioned in the lateral olfactory tract (LOT) and layer 2 for afferent and associational fiber stimulation, respectively. A recording pipette filled with ACSF (resistance: 4 MΩ) was then positioned above layer 2. Stimulation consisted of brief (0.1 ms) current pulses (10–100 µA). Afferent and associational stimulation was separated by 0.5 s. After obtaining a baseline of field excitatory postsynaptic potentials (fEPSPs), EPSCs or EPSPs serotonin was applied to the recording chamber. ChR2-expressing axons in the aPC were photostimulated through the microscope objective using the epifluorescent illumination via an LED light source (Thorlabs, Germany). Light intensity was set to 0.5 mW. Photostimulation consisted of a 3 s train of 10 ms pulses at 10 Hz. Control and photostimulation trials were intermingled. 

For the selective stimulation of DRN 5-HT neurons in vivo, we used similar protocols to the ones in Lottem et al. (2016). Briefly, SERT-cre mice previously (4–8 weeks) injected with 0.5–1 μL of AAV2/1–Flex–ChR2–YFP (AV-1-20298P, University of Pennsylvania, 10^13^ GC/mL) in the DRN were anesthetized with urethane (1.2 g/kg) and mounted in a stereotaxic frame. aPC neurons were recorded with a glass electrode (impedance: 8–20 MOhm) filled with saline and connected to a DC amplifier (Axoclamp 2B, Axon Instruments, San Jose, CA, USA). Electrophysiological data were acquired using a Power 1401 and Spike2 software (Cambridge Electronic Design, Cambridge, UK) and stored on a personal computer for offline analysis. Spike sorting was performed using Spike2 software (Cambridge Electronic Design, UK). An optical fiber (200 µm diameter; numerical aperture 0.38) was inserted above the DRN for photostimulation (a 5 s train of 10 ms pulses at 30 Hz, 5 mW). The 5-HT_2B_ receptor antagonist GR127935 (3 mg/kg, dissolved in saline) was administered intraperitoneally. Statistical significance was assessed by Student’s t-test, and a *p* value below 0.05 was considered significant.

## Data Availability

Data are available upon reasonable request from the corresponding author.
